# The New York Institute of Stomatology

**Published:** 1899-09

**Authors:** 

**Affiliations:** The New York Institute of Stomatology


### Reports of Society Meetings.

### THE NEW YORK INSTITUTE OF STOMATOLOGY.

A REGULAR meeting of the Institute was held on Tuesday evening, May 2, 1899, at the office of Dr. J. Adams Bishop, No. 30 West Forty-eighth Street, the President, Dr. E. A. Bogue, in the chair.

The minutes of the last meeting were read and approved.

The Secretary presented a letter from Dr. W. H. Trueman, associate member from Philadelphia, stating that he had recently devised a new and practical method of making seamless gold crowns, and that he had mailed to the President of the Institute samples of this work together with a paper of instructions.

### COMMUNICATIONS ON THEORY AND PRACTICE.

*Dr. Karl C. Smith.*—I have only a few words to say regarding a simple and very effective method of wedging teeth. It is a method which I have never seen described in dental literature, and, so far as I am aware, is not in common use in the profession.

The materials required are very simple,—namely, heavy linen or carpet thread of various sizes. A size is selected which will with difficulty pass between the teeth to be wedged; grasping the string at both ends, it is pulled down between the teeth until it passes the shoulders. The ends are then brought together and a knot tied at the side, thus pulling the cord against the shoulders and preventing it slipping down on the gum and causing irritation. I find this method is effective, and obviates a great deal of soreness, patients not complaining at all of the wedge.

*Dr. H. G. Marshall.*—This method is very similar to one which I have been using with success for several years, the only difference being that I use a silk thread and, in conjunction with it, a shred of cotton. I apply the silk in the manner which Dr. Smith has described, and before tying the knot pack between the teeth a few fibres of cotton. By knotting the loop of silk around this cotton fibre it is prevented from slipping down on the gum, and at the same time the cotton is so held in place that its expansion is more effective in separating the teeth between which it is applied.

The size of the cavity between the teeth must be taken into account and enough cotton applied to fill this. Care should be taken not to apply too much cotton. I have often separated teeth in this manner in twenty-four hours and without soreness.

*Dr. S. E. Davenport.*—If it had been my good fortune to devise the method of wedging of which I had expected to speak, I should be tempted to keep my seat after hearing the ground so thoroughly covered by Drs. Karl Smith and Marshall, but in justice to the gentleman who, so far as I know, first used it, I beg to be allowed to devote a few moments to the subject. This is usually spoken of as "the fish-line method," and I have supposed that the line was Chinese grass line, but our President informs me that it is in all probability silk.

Dr. Isaac B. Davenport, of Paris, France, nearly ten years ago noticed in the mouth of a patient, who had just landed from one of the steamers from Boston, a wedge consisting of a doubled silk tied in a manner similar to that described by Dr. Marshall, with cotton between. It instantly occurred to Dr. Davenport that fish-line, which he was then using for regulating purposes, would be very valuable also for wedging, and after experimenting with different sizes, he came to the conclusion that it was better to use the line without the cotton. The method is especially applicable to those cases where the teeth are snugly in contact at the masticating surface with a V-shaped space at the gum, and where it is almost impossible to apply tape, wood, or rubber wedges without having them slip down against the gum. To apply the fish-line a piece of doubled waxed floss silk should be passed between the teeth, leaving the loop sticking out either on one side or the other. Into this loop the fish-line, of the size decided upon, is threaded and drawn through. The two ends of the line are then tied with a square knot a little to one side of the masticating surface. Applied in this manner, there is no danger of the wedge working down to the gum, the knuckles of the teeth preventing.

While sufficient space for filling may often be gained in twelve hours with but little soreness, one line will continue swelling for two or three days, where extensive wedging is needed. The method has been of greatest service in my hands for many reasons, the most important of which is that the gum is not encroached upon, inflammation of the gum being, in my opinion, the principal cause of soreness in wedging. My experience in regulating teaches me that the teeth can be moved for a considerable distance without much soreness, provided the gum is not injured.

The possibilities of this method are very great, both for wedging and regulating teeth. I have, on various occasions, expanded arches by this method without using a plate, simply by placing these wedges between the teeth, although I believe that extensive expansion can better be accomplished in other ways.

One great advantage of this material over tape or cotton used in wedging is that it is twisted so tightly that it does not become offensive when left in the mouth for many days. The principal objection to the fish-line method is that the materials best adapted for this purpose are very difficult to obtain in the United States. I have found that the line obtained here does not seem to swell as much as that purchased in Paris.

I would say, in conclusion, that teeth with broad approximating surfaces in contact nearly to the gum are not favorable for wedging in this manner, as, in order to place the line in position, it is necessary to encroach upon the gum. For teeth of this character I think the use of tape would be preferable.

*Dr. C. B. Parker.*—It is possible to obtain this grass line of Messrs. Abby & Imbey, of this city, dealers in sporting goods. I have been obtaining a very satisfactory material from them for six or seven years.

*Dr. F. Milton Smith.*—I have been using for several years Dr.

Marshall's method, and wish to offer one or two suggestions about applying the silk. It is frequently found almost impossible to get the floss silk through by bringing it up from below. For a long time it has been my practice in these cases to take a very fine cambric needle, No. 9, draw the temper, bend it, and thread it with a fine silk, and to the silk attach the line or whatever is to be used in wedging. This needle is passed between the teeth from above next the gum; and the line drawn through in this manner. Again, instead of using a line of large size, I generally use a small size, making it into a chain stitch.

*Dr. Marshall.*—I think Dr. Smith's suggestion regarding the cambric needle a very good one. It is a method which I have been using, in a modified form, for some time. I take a No. 9 needle and grind off the head to about the middle of the eye, leaving a little pitchfork. I also find this very useful in carrying dressings into a canal.

SPECIAL TOPIC.

Discussion of Dr. F. Milton Smith's paper, read before the Institute last November and published in the March number of the INTERNATIONAL DENTAL JOURNAL, on the subject of the immediate filling of the roots of pulpless teeth:

*Dr. F. Milton Smith.*—When I read my little paper last November and finished about ten o'clock, with no time for discussion, I began to congratulate myself that my paper would be left to that condition described by one of our ex-Presidents as one of innocuous desuetude.

I will briefly call attention to some of the principal points of the paper which I would like discussed, and will read from the reprint, on the ninth page:

"In summing up, we would say that we regard as essential to success in root treatment, first, the rubber dam; second, free direct access; third, thorough cleansing mechanically and antiseptically; fourth, getting the antiseptic through the root; fifth, perfectly filling the root, immediately upon getting an aseptic condition, with an antiseptic root-filling; sixth, sufficient confidence in the method used to insure thorough work, and the minutest attention to details.

"Given these conditions, we believe that any careful operator who treats and fills roots at once will succeed far beyond the one who keeps his roots under prolonged treatment. . . . We have a theory that to very carefully open a root and remove two-thirds of the canal contents after bacteria have been awakened in the other third and beyond the root, is to invite the trouble we seek to avoid. On the other hand, we believe that where we have just opened the tooth, and set forces at work which are bent on its destruction, we ought at once, if possible, to get our antiseptic not only in the root, but also as much farther as the mischievous effects have gone. After we have done this, and before more micro-organisms have come to life, we ought to forestall them by filling the root so thoroughly with an antiseptic material that there will be no home in which they may abide."

I believe this fairly covers the claims which I made as to the desirability of filling roots at the first sitting.

*Dr. C. O. Kimball.*—I think Dr. Smith has a paper by Dr. Stockwell, a synopsis of which he is going to give us.

*Dr. Smith.*—In November Dr. Howe kindly called my attention to the fact that Dr. C. T. Stockwell, of Springfield, Mass., was the first to advocate the immediate disinfection and filling of root-canals. I wrote to Dr. Stockwell, suggesting that I had never read his paper, and would be very glad to hear from him on the subject. I received a very delightful letter enclosing the original manuscript of a paper read by him before the New York State Society in May, 1887. As the paper is quite long, I have made a synopsis, which Dr. Stockwell has read and endorses.

SUMMARY OF PAPER READ BY DR. C. T. STOCKWELL.

My treatment of devitalized teeth, with or without fistulous openings, is based upon the theory that we have practically only a single agent to deal with,—namely, sepsis. In cases of acute pericementitis, I consider micro-organisms and the material favorable to their proliferation as the fact of first importance, and in proportion as I succeed in eliminating them lies my hope of relief. For this purpose I find peroxide of hydrogen an admirable agent, as this not only dissolves but throws out of the canal much of the disturbing matter.

In cases of so-called blind abscesses (while some have contended that we are not warranted in filling the root until we have a reasonable certainty that the abscess is destroyed and the process of repair has begun, hence a long time must intervene before filling) I feel that we are warranted in filling the root whenever the proper conditions are obtained, even though it may take but a few minutes to secure them. The same holds in acute cases: first remove the putrescible matter and destroy the septic agents, then nature will take care of the case if the entrance is closed so that more septic influence may not enter.

It has been suggested that the cleansing of pyogenic tissues and the application directly to them of antiseptic medicaments is of itself no assurance that the suppuration will not continue, and we are not justified in proceeding as if it had actually been arrested and repair begun.

I wish to state most emphatically that I do not experience the trouble here apprehended, although I do proceed on just that assumption. The peroxide of hydrogen having eliminated the troublesome matter from the pulp-canal and the tubuli, also reaching and cleansing the pyogenic tissues, the root being filled with a non-irritant and powerful antiseptic material, the subsequent deposit at the apex of the root of amœboid cells or inflammatory exudates would not by any means necessarily be the source of further offence to the surrounding tissues.

If the condition we are called upon to treat is due to organisms or spores that have found entrance, it seems reasonable to suppose that if they are eliminated nature will repair the tissues by readily organizing the amœboid cells into normal tissue. The inflammatory exudate will take care of itself if the pyogenic fungi are excluded.

Professor Black states that the production of pus is an act of pyogenic fungi which takes advantage of a definite form of opportunity. I am convinced that peroxide of hydrogen, bichloride of mercury, iodoform, and eucalyptol not only destroy organisms of fermentation and putrefaction, but also pyogenic fungi. I therefore feel that after using the first two there is a great advantage in combining the latter in the root-filling.

After treating hundreds of cases by a method adopted two years since, with no failures, I am ready dogmatically to assert that a very large majority may as well be treated and filled at a single sitting. I consider the non-action of peroxide of hydrogen as almost a sure indication that the root may safely be filled.

Briefly I will state my method. First, remove all *débris* as thoroughly as possible, repeatedly inject peroxide of hydrogen, waiting each time till bubbling ceases, then absorbing it and applying more until not a single bubble appears; if a fistula exists get the remedy through it. Next, dry the cavity thoroughly and flood canals with bichloride of mercury 1 to 1000; after a few moments remove the surplus and thoroughly bathe with Sander & Sons' eucalyptol, making that the vehicle for a considerable quantity of iodoform. I then dip fine points of gutta-percha into a solution of eucalyptol and iodoform and fill the roots with them. I consider it very important to get Sander & Sons' preparation of eucalyptol. I do not use carbolic acid, and believe that, owing to its coagulating effects, it materially limits the usefulness of the other remedies.

I have letters from dentists who have treated teeth for the past year giving detailed accounts of their work, in which all agree that the method is a success in their hands. I attribute the good results, first, to the cleansing action of peroxide of hydrogen, and, secondly, to the strong and persistent antiseptic properties of the root-filling. Were I to leave the iodoform and eucalyptol out of the filling, I should not expect to succeed as I do.

*Dr. Marshall.*—Some years ago I had the good fortune to have my office in the same house with Dr. Smith. During this time a patient came to me by recommendation, with a left superior lateral incisor badly abscessed, without a fistulous opening, and a history of having been treated by a prominent dentist for over three months without success, the abscess being just as bad as at the beginning, discharging pus through the canal at every treatment. Dr. Smith had often talked with me about his method of immediate filling but, as I was rather sceptical, I carefully treated this tooth and left a dressing of carbolic acid in the canal. The next day she returned with the face and gums badly swollen, and the dressing had to come out. I talked the matter over with Dr. Smith, and as a result I treated the case after his method and immediately filled the canal. Two weeks hence she had had no trouble, and I then filled the tooth with gold.

Some years ago a lady came to my office with an upper central incisor which had an abscess with a fistula, and which had been discharging pus for many years. At that time I cleansed it thoroughly, pumping several antiseptic solutions through it and removing a quantity of pus. The next day she came back and insisted that the tooth be immediately filled, saying that she would not submit to treatment, as it made her mouth sore. I washed it out as thoroughly as I could with carbolic acid, and filled it with oxychloride of zinc. The abscess healed and never returned.

*Dr. D. A. Fuller.*—I have been very much interested in Dr. Smith's paper, although I was not at the November meeting. If one-half of what Dr. Smith says is true, I think we will all soon be following in his footsteps. There occurs to me, however, one or two objections. In a case, for instance, of acute pericementitis, the patient often comes for immediate relief, and while it might be possible to devote fifteen or twenty minutes to the case at that time, it would not be convenient to devote the time necessary for the thorough treatment and filling as advocated by Dr. Smith. Again, the length of time required—from an hour and a half to two hours—might prove wearisome to patients in this condition.

In regard to Dr. Stockwell's paper, I would say about peroxide of hydrogen, that I think I should go rather carefully with this agent if there was much of an opening at the apex. I can speak from personal experience, as I have had it tried on myself. I do not think I ever suffered a couple of moments of quite such severe agony as when the peroxide was applied to one of my teeth then under treatment. In my hands oil of cinnamon and wood creosote have proved rather better than carbolic acid.

*Dr. J. G. Palmer.*—I do not like to deliberately contradict Dr. Smith, the essayist, but it seems to me that there are some discrepancies in his statements which cannot be overlooked. On the fourth page of the reprint he states, that in order to feel absolutely safe it is necessary that a fine broach be passed through the apical foramen, "for then we feel quite confident that our remedy will reach the proper place." Again he states that "there are many canals which it is impossible to enter with the finest broach, and which we do not fill except as we can force a little of our solution into them." My inference then is, that there are some teeth which he would fill at the first sitting without going through the apical foramen. I think there are some other things to be taken into consideration besides the condition of the tooth itself and its immediate surroundings. We should carefully take into account the health of the patient. It is good practice and it has been my custom to fill the roots of pulpless teeth at one and the same sitting in cases where I have thought the health of the patient permitted it. But this treatment of a tooth with exactly the same local surroundings and conditions, but where the general health of the patient was poor, might result unsatisfactorily.

Again, I do not think the majority of our patients would care to submit to the "from one to four days' soreness" which Dr. Smith admits is quite likely to follow his method, sometimes even swelling of the face. It seems to me in his paper that he has not discriminated sufficiently between the cases which he would fill immediately and those which he would not, and this method in the hands of a younger practitioner might result disastrously.

I think I can illustrate my idea by a case which I treated recently. A boy twelve years of age, when nine years old fell upon the ice, breaking off his two superior central incisors. The roots were treated and filled, apparently successfully until six months later, when fistulæ formed over both roots. Without removing the fillings the dentist who had the case in charge treated these teeth for three years through the fistulæ. Finally one fistula closed entirely, while the other, the left one, remained persistent. Later there appeared in the roof of the mouth a large swelling over the root of the right central, which finally discharged and disappeared.

When the child came into my hands the fistula on the gum existed, but the swelling in the roof of the mouth had entirely disappeared and there was no soreness. I removed the fillings from the roots and dressed the canals with carbolic acid and oil of cloves. Some time within a week the swelling appeared again, but did not discharge. I removed the dressings and syringed with the three per cent. medicinal pyrozone, forcing it up into the tissues. It caused a little pain at the time, but in twenty-four hours the swelling had entirely disappeared. I cannot help but feel that if I had in this case followed Dr. Smith's method, and filled the roots after treatment at the first sitting, the result would have been a swollen face, and I should have had to remove the fillings. I cannot see how I would have been any more successful than the man who first filled the canals, as the gutta-percha fillings which I removed were apparently perfect. My experience with Dr. Smith's method in my first case was so unsatisfactory that I have been inclined to be a little cautious. The case was that of a young dental student, who presented a superior first bicuspid which was discharging considerable pus through a fistula. The tooth was filled immediately. Within the next twenty-four hours his eye was nearly closed, and before I could get the dressing removed the student had the tooth extracted.

I should also like to have Dr. Smith draw the line as to how sore a tooth must be "to be too sore to operate upon." Recently, in the case of a brother dentist, a left superior second molar presented having in each canal a very large foramen. The tooth was somewhat tender to occlusion, and even the suction caused by withdrawing the dressing caused pain. Treatment by carbolic acid and filling at the third sitting was followed by no soreness. I am confident that if this case had been treated by Dr. Smith's method, it would at least have been one of the cases which would have been followed by considerable soreness. The patient is a very busy man, and coming to me at the close of a long day of arduous work, I felt that much time and care were needed because of the depressed condition, hence did not fill immediately.

*Dr. C. C. Linton.*—I have been practising this method of immediate root-filling almost exclusively about as long as Dr. Smith, and with excellent success. I counted up at one time, and found that I had not averaged a loss of one tooth in a hundred by this method. My treatment is about the same as Dr. Smith's, except that I use hydronaphthol in the solution instead of iodoform. I find this method also excellent in the treatment of children's temporary teeth, having recently treated and filled the four lower temporary molars at two sittings. I had a patient last week who had been suffering two or three weeks with an upper wisdom-tooth. I removed the dead pulp, treated and filled immediately, without adjusting the rubber dam, as the tooth was so broken down. The treatment was successful, and there has been no pain since.

*Dr. W. St. George Elliott.*—It is a great many years since we first commenced to hear of the advantages and the disadvantages of the immediate filling of the roots of teeth. Everybody has had a large amount of success and no one any failures. Ninety-nine per cent. of successes is very common. Our president knows very well of the German dentist who does not take the trouble to put on the rubber dam, does not trouble to remove *débris* from the canals, simply places in the canals a little of Witzel's paste, which is composed of thymol, oxide of zinc, and alum, and he can show ninety-five per cent. of successes. I think the whole thing depends upon the ability of the dentist to properly diagnose his patient, whether it is a proper case for immediate root-filling or not.

I am somewhat familiar with the methods of immediate root-filling, and have practised them. I had a case not long ago which I treated most thoroughly. I drilled it and Donaldsoned it; I used peroxide of hydrogen, oil of cassia, oil of cinnamon, hydronaphthol, and, finally, as the tooth seemed in perfect condition, I filled the roots with oxychloride of zinc, yet I was not at all surprised when I had to take the filling out and resume treatment. The patient was not in the proper condition, as I learned afterwards.

*Dr. C. G. Pease.*—In filling root-canals I am also guided by the conditions of general health of the patient. I have cured abscess at one sitting. I remember one case in particular, where, after evacuating a large amount of pus, I pumped in the peroxide of hydrogen and filled the canals immediately. Two years afterwards I saw the patient, and there had been no further trouble. I should like to ask what success has been had with the different makes of peroxide of hydrogen. I sometimes find it to be very poor.

*Dr. T. W. Onderdonk.*—The only solution I use in the way of peroxide of hydrogen is that prepared by the Oakland Chemical Company. I find it permanent, and with it I get excellent results. Regarding the immediate filling of roots, in my own practice I do not see the desirability of it, especially where we are seeing our patients every week, except in cases of fistulæ, where I always fill at once after a thorough disinfection.

*Dr. C. O. Kimball.*—I have a word to say on one point, and I will relate my experience in a case which it seems to me will be of interest. It is apropos of carbolic acid. I had under treatment some years ago an abscessed lower first molar tooth. The patient, who had been abroad for some time, returned with this state of things. For some reason or other this tooth resisted all my efforts to heal the abscess. I treated it with carbolic acid and with wood creosote without success. I made sure that I was getting in a sufficient supply of the drug. I had the tooth smelling very strongly of creosote, still the abscess kept discharging. At last I noticed that the irritation seemed to be the greatest immediately after making the application. It then occurred to me that perhaps we had all the antiseptic effect from the creosote that we could hope for, and that we were now getting an irritating effect which we did not want. I therefore washed it out very carefully with water and then with alcohol, and after drying the canal I treated it with carbolic acid, only five per cent. There was no reaction from this, and within a week the tooth was in an entirely different condition.

Since that time I have very seldom used pure carbolic acid in the treatment of teeth. I think we get quite as satisfactory antiseptic results with the weaker solutions; one that can be placed in the mouth without an eschar, and consequently one which does not cause irritation of the tissues.

Regarding Dr. Smith's method of treatment, it seems to me that while there are probably many cases where the immediate filling would be indicated, there are many other cases where it would not do at all. In those cases which come to us when the tooth is very sore and tender, with an active state of inflammation existing, I

would not dare to follow this method. The first indications, it seems to me, would be to relieve the inflammation and get the tooth in a quiet state, keeping it closed and sterilized meantime.

There is another point to which I would like to call your attention, which has been useful in my practice. I make it a rule to measure the length of roots of each dead tooth in thirty-seconds of an inch, and record the measure upon my chart, so that in subsequent treatments I know exactly how far it is safe to go. I also use marked instruments, so that there is no possibility of irritating the tissues beyond the apex. A very simple way of marking an instrument is to touch it with a stick of hot shellac, forming a tiny bead, being careful not to heat the instrument enough to draw the temper. This bead catches the eye and enables me to tell just how far my instrument has passed into the canal.

As to the method of filling roots, it seems to me that the plan of using semifluid substances which are very easily forced into the tissues beyond the apex is defective; the oxychloride of zinc, for instance, which makes a very satisfactory canal filling in every other respect. For this reason it has seemed to me wiser to close up the apex by a substance which shall be permanent and prevent the filling from passing through the foramen. To accomplish this I carefully mark the length of the canal, and, using an instrument that is cut squarely across the end, carry a little bit of gutta-percha or gold to the apex.

A very good way to sharpen an instrument on the end in this manner is to pass it through a pore in a hickory block which has been cut squarely across the grain, and then grind off on a stone. Under a magnifying glass this shows a sharp clean edge all round.

My habit is to use the gold at the apex with oxychloride of zinc. By this method of measuring instruments the work can be made quite positive.

*Dr. E. S. Robinson.*—In the treatment of root-canals I generally use campho-phénique. It has great antiseptic properties and very little irritating effects, and I find it very successful. I generally stop the apical foramen with a pellet of gold.

*Dr. Pease.*—I think it is generally assumed that every case is not a proper one for immediate filling. I do not think that even Dr. Smith will claim that this can be done in every case. I have been using the method for three years, and I can say that I have had no failures, but by that I do not mean that I have filled in this manner every tooth which has come under my care to be filled, and

I believe it has only been by a judgment in the selection of cases that has brought about this success. I think we must expect some soreness in the treatment of canals in every case, and immediate filling is generally followed by soreness, although not very severe. Where the pain and soreness is so severe that it cannot be tolerated, I find nothing better than cold applications in the mouth. I start with luke-warm water, allowing a piece of ice to dissolve in it until the patient can no longer tolerate it. Of course, there are some cases where heat is indicated. This pain, once relieved in this manner, will not recur again.

*Dr. L. C. Leroy.*—In the case mentioned by Dr. Palmer of the two central incisors, it seems that that is a case where judgment should be used. Dr. Palmer may never expect to cure that class of cases. The apical end of the root was probably never perfectly formed through interrupted development, and I do not think, in those conditions, we can expect a permanent cure.

There will probably be subsequent recurrence. I should certainly expect trouble in a case of this kind, and would tell the patient so, and why. Again, in cases where we are not able to get the canals perfectly dry we cannot expect success. We must always, if possible, get to the end of a root with our pulp-canal filling. It has been stated that in some instances we do not and cannot get to the end of the pulp-canals. Such a filling is defective, and must be entered upon our ledgers and on the charts as an imperfect operation. Surgeons in any other branch of the healing art recognize that certain conditions may present in their work which are practically insurmountable and preclude denominating that particular case as a perfect operation. Just so with root-canal fillings. The unfilled portion acts as a closed sac. Serumal exudate, with the pyogenic white blood-corpuscle, will occupy the territory. There may be no evidence of defect for some time, but some day when that patient's physical tone is low, there will be a lameness about that tooth.

*Dr. Davenport.*—Dr. F. Milton Smith will pardon me if I say that in some things besides the filling of root-canals he is also an extremist, but as between the extremes of filling roots at the first sitting and continuing the treatment over a long period of time, give me the method which has been so ably championed by Dr. Smith. It is by the efforts of such men that all professions make their progress.

There is, however, great danger from the use of these extreme methods in the hands of a considerable proportion of operators. Of course, Dr. Smith, with his careful and accurate ways, is successful in nearly all cases, and I have no doubt he uses the same judgment and discrimination which several gentlemen have mentioned as being so necessary to success, but there are many men who would not be successful, if for no other reason than this inability to discriminate between favorable and unfavorable cases.

The other extreme from Dr. Smith's method is the one used by many men who will carefully open into a tooth with a putrescent pulp, merely making a drill-hole into which they will pass a pellet of cotton with an antiseptic, carrying the cleansing process a little farther with each sitting.

My plan has been a middle course, removing all the *débris,* if possible, at the first sitting, rendering the canal mechanically clean. I then wash with warm water and dry thoroughly with absolute alcohol and hot air, which is one of the best remedies and one which I depend upon more than any drug at such times. I have often said that the majority of cases which come to me for treatment I would be willing to undertake without drugs, supposing, of course, that there is no fistulous opening. When there is a fistula I force pure carbolic acid through, if possible, and the medicament I usually prefer when there is no fistula is a five-per-cent. solution of formalin. After this thorough first treatment I stop tightly with gutta-percha, and if, as is usually the case, no trouble has resulted, I fill the roots at the second sitting with chloro-percha and gutta-percha points.

*The President.*—I have learned a great many things this evening. Still there are a number of things which have not been mentioned, and as Dr. Smith is to take the stand in answer to questions, I trust he will clear up some obscure points for us. He has not yet told us what teeth he is referring to, or their conditions. Patients come to us with pulps exposed: they come with pulps all gone, but with no soreness until they are opened into; they come with what we call blind abscess and quite sore; they come with every variety of abscess. After which of these conditions are we to fill immediately? These questions have come up so many times that I am desirous that Dr. Smith should answer them to our satisfaction and instruction.

The instruction which Dr. Smith gives, and with which I heartily agree, is that the roots should be thoroughly cleansed. Dr. Kimball sounded a note of warning which I think it is well to look into; it is the placing of a pellet of gutta-percha or gold at the apex of the root, but Dr. Kimball did not tell us whether this applies to the six front teeth or all the teeth.

Regarding antiseptics, I find formaldehyde very acceptable. Some gentleman spoke of the use of oil of cinnamon, but I would suggest that he should not use the oil of cinnamon in the front teeth, as it will discolor them.

*Dr. J. Morgan Howe.*—The immediate filling of these roots that have not contained putrescent pulps is a procedure to which no dentist, I suppose, will object. Neither will any disapproval be offered to the immediate filling of roots that have had putrescent pulps in them, after they have been disinfected, provided the vital tissues beyond the apex are so near to a physiological condition as not to need either restorative treatment or a rest.

But the question raised by the procedures Dr. Smith calls a "positive method" is on the desirability or propriety of filling a putrescent root-canal after such disinfection as may be accomplished in one sitting.

Dr. Stockwell, of Springfield, was, I believe, the author of this method, and I think he has stated that his first operation of the kind was performed for a patient who could give but the one time for it, and was under the necessity of immediate departure for distant parts. His testimony and that of our essayist, as well as of others, are amply sufficient to establish the fact that teeth do endure such treatment well, and that the results have been regarded by them as successful. But I wish to direct the attention of practitioners and advocates of this practice to the proofs of success offered by the advocates of filling roots with cotton saturated with some antiseptic,—a practice which Dr. Smith appears to condemn,—and to point out also that it often appears quite successful to leave roots entirely undisturbed, or to treat with some so-called mummifying paste, after the death of the pulp, when many such teeth remain for years without causing irritation. These methods are perhaps just as positive as that we are specially considering, but neither of them afford any ground for claiming absoluteness or certainty of results. I think no method, in so far as it affects the living periapical tissues, can be regarded as positive or absolute in freedom from risk of producing irritation.

My reason for recently reporting the unfavorable result of opening into a putrescent root-canal—to which Dr. Smith has done me the honor to refer—was that it was so remarkable and unusual to have any irritation whatever follow treatment with the precautions mentioned, and to have suppuration occur in so little time. I do not think it possible to remove the putrid matter from the apical third of most root-canals without great risk of forcing some of the infectious matter through the foramen. The results of such infection, if it happens, must always vary with the virulence of the toxines, the relative antiseptic qualities of the blood, and the vital resistance of the tissues of different patients. Of these, of course, we can know but little in advance. I regard the statement of Dr. Starr as an important admission, which other advocates of immediate root-filling have not heretofore made, I believe. His statement to patients, that they are liable to have trouble for a few days if the roots are filled at once, is in accordance with what would seem to be an inevitable consequence of possible infection of living tissues beyond the apex.

I should think the liability of such infection much greater when attempts are made to remove all the putrid matter from the root at once than if the most remote part of it was first rendered innocuous by disinfection. If we suppose the living apical tissues to be inoculated with a portion of the root contents, a pathological disturbance will not certainly be averted by the use of antiseptics, nor will the chance of its modification by treatment be so good if access to the irritable tissues is prevented by a filling in the root-canal. The recognition of unfavorable results following very active efforts to cleanse putrid root-canals has been so general among careful practitioners that the caution against it, and advice to disinfect first, has been quite common.

Our fellow-member, Dr. Shaffner, of Florence, is the most recent one to offer a caution in this direction. This he has done in a paper read before the American Dental Society of Europe, on "Some Hints on the Manipulation of Pulp-Canal Probes."

While I am very ready to admit that a good proportion of putrescent root-canals may be treated and filled immediately without creating disturbance in the tissues beyond, I do not think it is nearly so free from risk of producing such irritation as the method which is followed by those who seek to render the putrid matter in the finer portion of the canal innocuous before attempting to remove it, taking more time to disinfect later. In regard to thorough disinfection of putrescent roots, there can be no doubt that repeated dressing of canals with disinfectants is required to disinfect dentine thoroughly.

Dr. Black has shown that over twenty-five per cent. of the dentinal tissue is organic matter, and that nearly eleven per cent. is water. When such a proportion of the dentine as this is putrescent matter, it is safe to say that the application of a disinfectant to the openings of the tubuli during one sitting will of necessity be insufficient to affect their contents to much depth. Stopping in the disinfectant and repetition of its application are required to permit the interchange and reformation of molecules that should occur throughout the length of the tubuli, before the canal can be considered in the most favorable condition to receive a filling.

*Dr. Smith.*—I have been more than delighted with the discussion this evening, and if the gentlemen were not already tired out I should be very glad to speak at some length on the subject, as I have enough material to last me for some time. Dr. Fuller spoke of acute cases which are extremely sore. On the fourth page of the reprint, lines four and five, I say that we fill these roots at the first sitting, provided the tooth is not too sore to work upon. If the tooth is very sore I do not torture the patient, simply open the tooth and leave it.

Regarding Dr. Kimball's suggestion that he would close such a tooth, let me say that I invariably leave it open until I see the patient again. I do not leave these cases to another sitting because I fear to treat the tooth at the time, but because of the pain incident to the mechanical work at the time of operating.

Dr. Fuller speaks of the peroxide giving pain. Dr. Stockwell claims that pain rarely follows the use of a reliable preparation.

I am pleased with Dr. Palmer's discussion, but sorry to learn that he has fallen from grace. I had supposed that he was converted two or three years ago. He states that I consider it essential to go through the foramen. I will quote from my paper: "Reference has been made to Dr. Flagg's caution not to go through the foramen. Regarding this we would say that we never feel absolutely safe unless we have gone through with a fine broach, for then we feel quite confident that our remedy will reach the proper place."

The fact has been lost sight of in the discussion that at no time have I stated that in every case would I fill immediately. As much is implied in the notice on your invitation, "It is almost invariably good practice to fill the roots of pulpless teeth," etc. Almost invariably does not mean in every case. It is not those gentlemen who fill teeth at one or two sittings that I am trying to help; it is those who are dressing and treating teeth time after time. I do not believe this is necessary.

The case mentioned by Dr. Palmer of the boy nine years of age may have been one of the exceptions, although I am very much inclined to think that I should have thoroughly cleansed and filled immediately. Regarding the swelling in the roof of the mouth, I think it should have been lanced. I lance all such cases. The reprint says, "We regard as essential to success sufficient confidence in the method to insure thorough work." Unless a man has confidence he will fail; that is, he will remove his filling when the patient comes back with a little pain or soreness.

In severe cases connected with the eye it has been thought good practice to inject a little pus into the eye to get up an active inflammation, and thus carry off the disturbance, and even gonorrhœal pus has been used. I think the irritation which we get with carbolic acid acts in a similar way. The suggestion has been made that the condition of the patient should be taken into consideration, and there are certain conditions without question where immediate root-filling is contraindicated, but these general conditions are not very prevalent. Dr. Elliott suggests that perhaps the claim of ninety-five per cent. of successes is not well founded. I have not claimed any percentage of success. All that I say is, I do not believe in the last ten years, where I have gotten to the end of the root and filled thoroughly, that I have lost three cases, and I do not believe that my failures go elsewhere.

Dr. Onderdonk suggests that the method is not necessary where the patient is coming every week. Confidentially, from a financial point, the method is not so successful. When I spend two hours over a patient and end by filling with gutta-percha, the patient is generally disappointed with the charge, exclaiming with surprise that Dr. So-and-so "treated my tooth for three months and did not charge me half so much."

Regarding the questions of the president, we are dealing, as I understand, with pulpless teeth, and I see no reason why teeth with exposed pulps or teeth with pulps partially devitalized should be brought into consideration here. In the cases with which the paper deals, I should fill immediately if I believed that I had gotten an aseptic condition.

Dr. Kimball suggests that he fills the ends of the roots with gold. Now I think it would be as reasonable for me to ask Dr. Kimball if he fills every minute root with gold as for some of the questions which have been asked to-night concerning passing a broach through the end of the root. Of course, he fills those teeth with gold at the apex which he can fill.

Regarding Dr. Howe's suggestion in reference to roots enduring in considerable numbers the treatment laid down. He is willing to admit this, but claims that the practice of filling roots with cotton saturated with an antiseptic, or even of leaving some roots entirely unfilled, may just as fairly be called a positive practice.

As to the cotton root-filling class. If they have confidence in their treatment, and have a method whereby they can pack the cotton sufficiently tight to make a thorough and permanent antiseptic root-filling, and do it with the expectation of having success in almost every case, we should consider their method a positive one. If, however, they use the cotton simply because they have no confidence with their treatment, expecting to remove it every time the patient comes in complaining of soreness, we should consider it anything but positive treatment. The practice of leaving roots unfilled we should consider nearly as positive as cotton put in after the latter plan.

Dr. Howe claims that none of these methods afford ground for absoluteness or certainty of results. We had supposed the paper would clearly set forth our idea that by a positive method we meant, not that in every case we succeed, but in every case we undertake the treatment with the expectation of success. This we think is more nearly a positive method than that vacillating one which is constantly expecting failure.

Dr. Howe also refers to Dr. Starr as being the first of the advocates of immediate root-treatment to suggest that patients are liable to have trouble for a few days. By reference to the paper of the evening of November 1, it will be seen that the essayist calls attention to the fact that patients are almost invariably warned that trouble of more or less severe nature is likely to ensue after treatment. In one of the cases cited the patient had some disturbance for several days. Regarding this I would say that I have never known a surgeon of any repute to refuse to use what he considers the best method of operating because his patient might suffer some temporary pain after the operation. In the aggregate we believe that our patients do not suffer anything like as much pain as when we employed the repeated dressing method, while we save more teeth and many hours of time to our patients and to ourselves.

Again, Dr. Howe says that the recognition of unfavorable results following very active efforts to cleanse putrid pulp-canals has been so general among careful practitioners that the caution against it and advice to disinfect first have been quite common. That the best and most careful operators do have much trouble with these cases we are willing to admit; in fact, we made this statement in the paper, nor have we anywhere intimated that we have no pain attending our treatment.

We are most careful to do thorough work. We do get up in many cases apical inflammation, just as Dr. Howe and the other disciples of continuous or repeated dressings do, but with this difference: we have confidence that the thorough work done, after the irritation subsides, will leave a useful tooth thoroughly filled to the end of the root and not likely to give subsequent trouble.

Dr. Howe goes with us as to the careful thorough work, also as to getting up the irritation, but there we part company. He does not have the confidence to say to his patient that the irritation is a necessary accompaniment to the treatment and will soon disappear, but removes his dressing, sometimes getting immediate relief and sometimes not. When he does get it, he has an open pulp-canal which is, as we believe, very quickly filled with germs of the same nature as those he effectually destroyed, which it would appear to us is coming back to the starting-point with no better chance of success the second time than the first.

We think that the fact of iodoform being incorporated in the root-filling may have a more beneficial effect in the way of combating septic influence than is generally supposed. This is in accordance with Dr. Stockwell's suggestion as we understood his remarks. So far as we know, Dr. A. J. Reinhold, in the *Independent Practitioner* for August, 1884, was the first to suggest this combination with chloro-percha.

I thank you very much, gentlemen. I still have faith in immediate root-filling, and shall continue in the practice of this method, because with it I have had success, and it is exactly the method I should wish practised upon myself under the same conditions.

It was moved and carried that a vote of thanks be extended to Dr. Smith for his excellent presentation of the subject.

Adjourned.

FRED. L. BOGUE, M.D., D.D.S.,
*Editor The New York Institute of Stomatology.*

